# Empowering informal caregivers of people living with dementia and enhancing digital health literacy through an extended digital intervention of the WHO *iSupport* manual: protocol for the project *iDEM-Support*

**DOI:** 10.1186/s12877-026-07123-4

**Published:** 2026-02-09

**Authors:** Nora Berner, Theresa Sophie Busse, Jan P. Ehlers, Franziska Anushi Jagoda, Amelie Meibeck, Ina Carola Otte, Julia Nitsche

**Affiliations:** 1 Faculty of Health, Chair of Didactics and Educational Research in Health Science, Witten/Herdecke University, Witten, Germany; 2 Faculty of Health, Professorship for Digital Health (Junior), Witten/Herdecke University, Witten, Germany; 3 Department of Health Services Research, Faculty of Medicine, Institute for Diversity Medicine, Ruhr University Bochum, Bochum, Germany

**Keywords:** Digital intervention, Individual education, Digital health literacy, Participatory design, Mixed-methods, caregiving support, Empowerment, Informal caregivers, Dementia, Accessibility

## Abstract

Informal caregivers of people living with dementia (PlwD) play a central role in home-based care. They are often exposed to emotional, physical, and organizational burdens, which increases their risk of psychological stress. Digital interventions have shown promise in supporting this target group. The WHO’s *iSupport* program offers a digital training platform for informal caregivers of PlwD; however, it has not yet been translated or adapted for the German healthcare context. Furthermore, deficits in digital health literacy (DHL) present a barrier to the use of digital health services. The *iDEM-Support* project aims to translate the platform *iSupport* into German, culturally adapt it, and develop a module to promote DHL. Using a mixed-methods design, informal caregivers and experts are involved in a participatory manner to enable user-centered development of both the platform and the DHL module. The content and design are developed based on qualitative interviews and focus group discussions with informal caregivers (involved in both steps) and experts (only involved in the focus group discussions). Usability is tested and evaluated through think-aloud sessions and internal testing by the project team. The project aims to empower informal caregivers by improving the usability and accessibility of *iSupport* within the German healthcare context. The platform *iSupport Deutschland* seeks to enhance the self-efficacy, digital competence, and psychosocial resilience of informal caregivers. Additionally, the project offers approaches for sustainable integration of the platform into existing healthcare structures. The *iSupport Deutschland* platform will be evaluated in a randomized controlled study following the research and development described here.

**Trial registration **Review: PROSPERO 2025 CRD420251048532.

## Background

In light of ongoing demographic shifts, which according to the Federal Statistical Office of Germany is characterized by a continuous rise in the population aged 67 and older [1], the prevalence of age-related illnesses – particularly dementia – is steadily increasing. Dementia currently represents the most common chronic syndrome among older adults: Worldwide, the estimated number of people living with dementia (PlwD) is around 55 million [2, 3]. In Germany, it is estimated that 1.8 million people live with dementia [4, 5]. Globally, approximately 84% of PlwD live at home and depend on support and care by unpaid informal caregivers – typically family members, friends, or other close individuals [6, 7].

At the same time, informal caregivers of PlwD frequently bear the full responsibility for care over extended periods – often spanning several years – while being exposed to a multitude of stressors, including financial difficulties, time pressure, and complex challenges in care management [2]. Empirical studies consistently show that informal caregivers of PlwD experience considerable substantial physical and psychological strain [8–10]. Compared to the general population, they face an elevated risk of developing mental health conditions such as depression and anxiety, as well as somatic illnesses including hypertension, gastrointestinal disturbances, and respiratory problems [11]. Moreover, informal caregivers of PlwD are themselves often of advanced age (frequently spouses) and therefore more likely to experience age-related health problems and multimorbidity [12]. This dual burden of managing their own chronic conditions while providing demanding long-term care further exacerbates psychological and physical strain and increases the risk of care-related overload [13].

When informal caregivers are no longer able to fulfill this role – due to excessive burden, exhaustion/burnout, or caregiving-related mental or physical health problems – the consequences for PlwD are often profound, particularly regarding to autonomy and the ability to remain in their preferred living environment. Chronic stress and burden can undermine the stability of a home care arrangement [14]. When the care situation becomes unsustainable, moving to institutional care is often considered. However, such a decision is not solely a consequence of the behavior of informal caregivers or potential conflicts [15], but rather, it is deeply connected to the overall fragility of the care network [13]. It is important to mention that relocating to a care home can also be distressing for PlwD. This transition, which involves an unfamiliar environment and the loss of familiar routines, can lead to a worsening of dementia-related symptoms such as anxiety, and decline in cognition and psychological well-being. This highlights the need to empower informal caregivers, as they play a central role in shaping both the daily lives and care trajectories of PlwD, and frequently act as their primary decision-makers. Supporting informal caregivers with adequate resources and interventions is essential to maintaining stable home care arrangements, delaying or preventing premature institutionalization, and improving the quality of life for both informal caregivers and PlwD. These findings underscore the urgent need for accessible and effective support interventions tailored to the needs of informal caregivers.

Digital educational interventions and programs [16] offer promising potential to empower informal caregivers: they can be accessed irrespective of location and time, are potentially scalable, and have demonstrated cost-effectiveness [17]. Over the past decade, interest in internet-based information and communication technologies for PlwD and their informal caregivers – including online training and support programs – has grown substantially [18]. National and international studies demonstrate that digital and face-to-face interventions have equivalent effects on informal caregivers’ emotional well-being, stress, and caregiver burden [19], risk of developing mental health disorders, and symptoms of anxiety and depression [20]. Moreover, informal caregivers report a reduction in feelings of being overwhelmed [20] and increased self-efficacy and confidence in caregiving tasks [15]. Digital formats also eliminate barriers such as long travel distances, limited professional resources, and concerns about stigma. In addition, they offer flexible access independent of time and location, allowing users to engage with content at their own pace and convenience. This reduces scheduling pressure, eliminates the need to arrange alternative care, and enables participation in familiar environments.

However, the mere availability of digital offerings does not suffice to engage target populations effectively. In Germany, significant deficits persist in the handling and utilization of digital health information [21]. There remain major societal challenges regarding digital health literacy (DHL): 75.8% of the German population exhibit low DHL (self-evaluation) [18]. DHL is defined as the ability to find, understand, evaluate, and apply relevant health information in digital environments [22] and is composed of traditional literacy, information literacy, scientific literacy and media literacy, computer and health literacy [23, 24].

In Germany individuals with lower DHL use digital health resources significantly less often [18] and are at risk of misinterpreting or being unable to verify the validity of health information obtained online. Previous studies and national health literacy surveys have shown that improving DHL is a significant challenge, and that it contributes to overall health literacy [18, 25, 26]. These findings highlight the need for promotion and training to encourage the competent use of digital health information. Furthermore, recent reviews have shown that informal caregivers, in particular, demonstrate high acceptance of digital interventions [27].

Against this background, the World Health Organization (2019) developed the digital training and support manual *iSupport* [2]. This generic, English-language intervention provides guided education, skills training, and social support to informal caregivers of PlwD, who experience stress, burden, and mild to moderate symptoms of depression or anxiety. This intervention empowers them to manage the care situation [17]. To ensure worldwide accessibility, *iSupport* must (1) be translated into the specific national language – given that many informal caregivers are older and may not speak English – and (2) undergo cultural adaptation to the local implementation context – to tailor content, examples, and values to the realities, needs, and expectations of informal caregivers. This process, guided by the WHO’s adaptation framework [2], has been completed and positively evaluated in randomized controlled trials in Portugal [28], India [29], the Netherlands [30], Greece [31], Spain [32], and Australia [33]. Although a German-language platform has already been implemented in Switzerland [34], a corresponding translation and cultural adaptation has not yet been realized in Germany.

While existing implementation research on *iSupport* has demonstrated positive effects, studies have also identified several barriers for its use, including lack of mobile accessibility, insufficient content personalization, limited moderation, and scarce peer exchange opportunities [28, 30, 33]. Low awareness of digital interventions [29] and low DHL further exacerbate these challenges and require targeted addressing. The project systematically addresses these findings.

### Study aim

The *iDEM-Support* project aims to translate the WHO’s *iSupport* into German and adapt it to the German healthcare context, implementing it as a digital learning platform – web-based, responsive and user-friendly. The translation is based on the existing German-language version of *iSupport*, which was developed for Switzerland [34], rather than translating the original English version. Although this version provides a high-quality linguistic foundation, it cannot be transferred unchanged to other German-speaking contexts. Despite the common language, there are relevant differences in terminology and usage, as well as differences in the healthcare system, legal framework, and available support services. Consequently, the Swiss version incorporates context-specific cultural references that may not be comprehensible or relevant to users residing in Germany. To ensure clarity, cultural relevance, and practical applicability for informal caregivers in Germany, the content will be systematically adapted both linguistically and culturally, including specific information and examples related to the healthcare system.

**Fig. 1:**
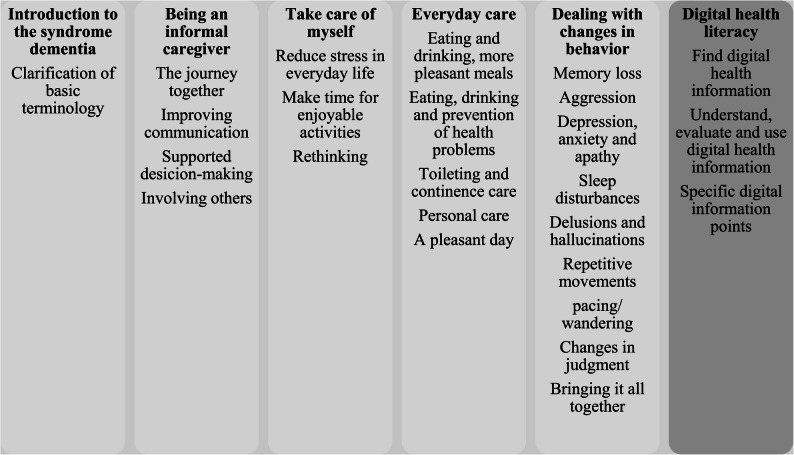
Content of the iSupport Deutschland platform (own representation). The figure illustrates the structure and thematic modules of the iSupport Deutschland platform. Modules 1–5 correspond to the core content of iSupport, module 6 introduces DHL, aimed at enabling informal caregivers PlwD to find, understand, evaluate, and apply health information digitally. The platform is web-based, responsive, and user-friendly, and includes interactive features, and learning objectives

The manual will be expanded to include a module on DHL tailored to the needs of informal caregivers of PlwD, as well as an interactive feature to facilitate interaction among informal caregivers and other users (Fig. 1).

The module on DHL is developed using a theory- and empirical-informed approach. It is grounded in an established conceptual model by Milanti et al. [35], who conceptualize DHL as a meta-competence that encompasses functional, interactive, and critical competencies required to effectively engage with digital health information and services. This theoretical framework provides a robust foundation for the systematic development, operationalization, and evaluation of learning content within digital health interventions. In addition to this theoretical framework, the development of the DHL module is informed by the findings of a systematic review (carried out during the course of the project) as well as by results from preceding empirical data collection, including qualitative interviews and focus group discussions with relevant stakeholders.

Building on this combined theoretical and empirical foundation, the module on DHL is designed to provide both foundational knowledge and practical guidance for effectively searching, understanding, evaluating, and applying health-related information in digital contexts. Specially, the module seeks to ensure that users know where and how to find reliable health information online, how to assess the quality and trustworthiness of digital health information, and how to use this information to make informed decisions appropriate to their individual life situations or to seek adequate support [23, 36]. The individual DHL components defined by the model of Milanti et al. [35] are systematically linked to specific learning objectives and interactive tasks. Depending on user needs, preferences and current behaviors in searching for health-related information via digital media, the module may include task-based learning activities that require the use of digital tools to promote engagement and competency development.

Furthermore, particular emphasis will be placed on ensuring conceptual alignment with the existing *iSupport* content whereby interactivity and usability are particularly important. We refer to the resulting platform below as the *iSupport* *Deutschland* platform, which can be seen as a further development of *iSupport*.

The *iSupport* *Deutschland* platform is designed to reduce psychological stress among informal caregivers and to improve their quality of life. The development therefore focusses on usability, instructional design, participation, the enhancement of DHL, and social networking, based on findings from previous studies in other countries [28–30, 33]. This will be followed by a proof-of-concept in a randomized controlled trial (RCT). If the proof of concept is successful, the *iSupport* *Deutschland* platform will be docked onto existing networks (e.g. Last Aid Germany gGmbH) and made permanently accessible as permanent links and applications on the websites of interest groups (e.g. German Alzheimer Society e.V., We care – Interest group and self-help group for family caregivers e.V. (Wir pflegen NRW e.V.), and Last Aid Germany gGmbH).

This study protocol describes the planned work packages for translation, cultural adaptation, identification of needs and current behavior for searching for information online, development and piloting of the new module on DHL and the platform. The RCT will be reported separately in an independent study protocol.

The project aims to achieve the following specific objectives:

1. To identify what needs informal caregivers have when searching for health-related information regarding care for PlwD online.
2. To determine how a digital intervention to support informal caregivers of PlwD regarding informal care and DHL should look and function technically so that it is accessible to all users.
3. To develop a structured module for improving DHL, guided by the theoretical framework of Milanti et al. [35], including relevant content and instructional design, so that informal caregivers of PlwD can improve their own DHL by working through the module.

## Methods

### Study design

The research design of *iDEM-Support* follows a mixed-methods approach that integrates both qualitative and quantitative methods to address the study aims comprehensively. The usability (including accessibility) of both the DHL module and the overall platform will be systematically assessed. Central to the methodological framework is the continuous involvement of prospective users, which ensures that the ongoing development of the platform remains closely aligned with the specific needs and expectations of the target group. The sample size was guided by practical considerations, including expected recruitment feasibility within the study period, resource availability, and alignment with comparable feasibility and pilot studies in the field. However, the respective data collections are always considered in relation to data saturation, and appropriate adjustments are made to the sample size if necessary to ensure data saturation and diversity of perspectives.

**Fig. 2:**
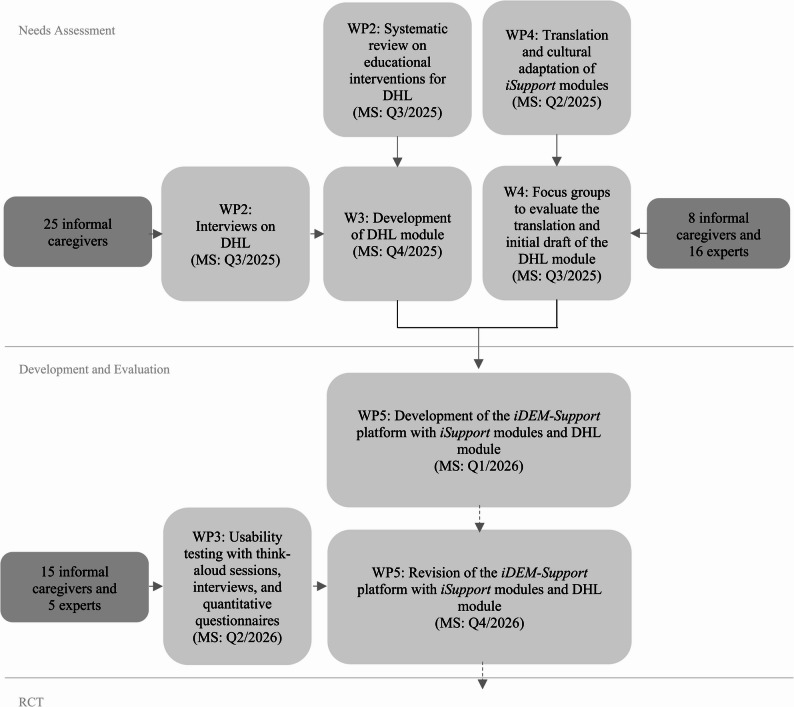
Applied steps in the *iDEM-Support* project (own representation). The figure shows the sequential work packages (WPs) and milestones (MSs) of the *iDEM-Support* project across the phases of needs assessment, development and evaluation, and preparation for a RCT. This process includes a systematic review, qualitative interviews and focus groups, development and cultural adaptation of the *iSupport* modules including a DHL module, platform development, and iterative usability testing. Milestones are indicated by target quarters (Q). WP = work package; MS = milestone; Q = quarter

The project proceeds in several interrelated steps (Fig. 2):

1. A systematic review will be conducted to identify the facilitating and hindering factors of digital educational interventions aimed at improving DHL among informal caregivers aged 18 years (age of majority in Germany) or older and older adults aged 65 years or above. The review question is structured according to the PICO framework, with informal caregivers and older adults as the population, digital educational interventions focusing on the enhancement of DHL as the intervention, no mandatory comparison group, and improvement in DHL as the primary outcome. People aged 65 and older were included because 65 traditionally marks the onset of retirement in Germany and remains a recognized legal and political threshold for older adulthood. Although the statutory retirement age is shifting toward 67, including individuals from age 65 ensures that relevant age-related changes are appropriately represented. Informal caregivers and older adults, often less familiar with digital technologies may also benefit from such interventions, but the primary focus is to understand how to best support informal caregivers and older adults in improving their DHL. Eligible interventions include web-based educational programs and mobile health applications designed to improving DHL. Interventional studies, as well as systematic reviews and meta-analyses, published in peer-reviewed scientific journals will be included. Only studies available in full text and published in German or English will be considered.
2. Qualitative semi-structured interviews with informal caregivers of PlwD will explore current behaviors and needs related to searching for health-related information in digital media. Participants’ DHL will also be assessed using the GR-eHEALS [37]. The GR-eHEALS is the validated German version of the eHealth Literacy Scale (eHEALS) [38], a widely used 8-item self-report instrument assessing individuals’ perceived ability to find, understand, evaluate, and apply digital health information. It has been used and validated in German-speaking populations, including older adults. In the *iDEM***-***Support* project, the GR-eHEALS will initially be used to assess baseline DHL and describe the study population. The measure will be re-administered following the intervention phases (Think-Aloud sessions and RTC), enabling the examination of potential improvements in DHL associated with use of the platform. Its focus on core DHL competencies directly aligns with the aims of the study and the learning objectives of the DHL module.
3. The translation and cultural adaptation of the *iSupport* modules as well as the initial draft of the DHL-module will be evaluated in focus groups with informal caregivers and experts. The aim is to ensure content accuracy, clarity, and usability based on user expectations and preferences.
4. The DHL module will be designed regarding content and functionality, based on the insights from the systematic review as well as findings from the semi-structured interviews and focus group discussions. These preparatory studies identified key challenges, needs, and contextual factors in the use of digital health information, ensuring that the module is evidence-based and closely aligned with the experiences and informational needs of the target population. Its instructional design is informed by the theoretical framework of Milanti et al. [35], current scientific evidence on effective didactical strategies, usability research, and content-specific findings in the field of DHL.
5. Think-aloud sessions with informal caregivers of PlwD and experts will assess usability-related challenges and inform participatory design. These includes (1) cognitive walkthroughs to document user interactions and thought processes, (2) post-session interviews for user reflection, and (3) standardized questionnaires – including the System Usability Scale (SUS) [39], the short version of the User Experience Questionnaire (UEQ-S) [40], and the GR-eHEALS [37] – to evaluate accessibility, usability, user experience, and DHL quantitatively.

### Participants

Informal caregivers of PlwD and experts are the study participants in the various data collections. Family members, partners, friends, neighbors or other close individuals are included as informal caregivers who provide unpaid, home-based care and support for a PlwD. This includes assistance with daily living activities, emotional support, coordination of care, and navigating health-related information and services. Experts in this context are individuals who have extensive experience with the perspectives of informal caregivers of PlwD and their situation – either from the perspective of self-help groups, associations, or professional expertise in research and care. Participants must be over 18 years of age, with all genders being considered. Only individuals who possess sufficient proficiency in the German language will be included. A diagnosed psychiatric disorder will serve as an exclusion criterion.

The samples and sample sizes in relation to the form of data collection are listed below:

- A total of 25 semi-structured interviews will be conducted with informal caregivers of PlwD, focusing on the topic of DHL.
- Three focus group discussions will be conducted to evaluate the translation and cultural adaptation of the *iSupport* modules. One group will include eight informal caregivers of PlwD, while two additional groups will each include eight experts. Informal caregivers and experts will participate in separate focus groups. This is to promote open and unbiased discussions. It is also to minimize potential hierarchically driven inhibitions.
- The think-aloud sessions to pilot the platform will be conducted with 15 informal caregivers of PlwD and five experts. The group of informal caregivers is divided into three subgroups: approximately five informal caregivers with high DHL, five with moderate DHL, and five with low DHL. Participant classification will be based on results obtained using the GR-eHEALS [37].

Participants will be recruited using a combination of digital and analogue recruitment approaches to enhance the inclusion of informal caregivers with varying levels of DHL. In addition to online recruitment via social media channels, newsletters, and official communications of the Department of Health Services Research at Ruhr University Bochum, the German Alzheimer Society e.V., We care – Interest group and self-help group for family caregivers e.V. (Wir pflegen NRW e.V.), and Last Aid Germany gGmbH, analogue recruitment pathways will be deliberately intensified. Printed project flyers and calls for participation will be disseminated through the affiliated member societies, which will be explicitly asked to approach potential participants directly. Snowball sampling will be strategically employed to specifically reach informal caregivers with lower DHL. Participating caregivers with higher DHL will be encouraged to share study information within their personal offline networks (e.g., self-help groups, caregiver support groups, dementia cafés), thereby facilitating access to caregivers with lower digital affinity. Additionally, a press release issued by Ruhr University Bochum was disseminated via regional media outlets. In particular, radio dissemination proved to be a relevant channel for reaching informal caregivers with limited digital engagement. Furthermore, the dissemination of study information by the technical product developer Ergosign GmbH enables access to individuals outside of traditional care-specific networks.

Overall, the combined recruitment strategy aims to mitigate recruitment bias toward informal caregivers with higher DHL and to improve the representation of informal caregivers with lower DHL, while ensuring a diverse study sample. Sample diversity will be actively promoted through the targeted recruitment of informal caregivers with varying sociodemographic backgrounds, genders, caregiving contexts, and levels of DHL. The key characteristics will be systematically assessed using validated questionnaire measures. This approach enables a transparent description of the achieved sample, a critical reflection on potential selection effects, and the ongoing monitoring of sample composition, allowing recruitment strategies to be adjusted if necessary to enhance diversity.

### Data collection

As part of the recruitment process, a contact list will be compiled containing basic contact information (name, address, email address, telephone number). This data will be collected via a contact form completed by prospective informal caregivers and experts during initial outreach. The completed forms will be returned to the research team by post or via e-mail. Prior to this, verbal consent will be obtained regarding data storage and inclusion in the contact list. For better understanding and traceability, data collection is structured according to the three interrelated steps:

- *Interviews*: In advance, sociodemographic data will be collected using a standardized paper-based questionnaire. Additionally, the GR-eHEALS [37] will be surveyed. The interview guides will be developed by the authors based on existing models of DHL and research findings on DHL. Following a narrative stimulus regarding the caregiving situation (“Please tell us about the care situation with your family member with dementia.”), the interview will first focus on participants’ experiences with digital health services (e.g. “Please tell us about a situation in which you experienced challenges when searching for health and/or care-related topics on the Internet.”). It will then explore their needs and expectations in the context of DHL (e.g. “What should a website look like or what should it contain to make it easily accessible and easy to use for you?”). Particular attention will be given to the technical features and devices participants use to access digital services (e.g. “Which digital devices (e.g., PC, tablet, smartphone) do you use, and which form of media (e.g., video, podcast, text) do you prefer?”). In addition, participants will be asked about their personal needs and preferences in engaging with the digital platform (e.g. “If a digital platform like the one we're planning enables communication with other informal caregivers, what form would be most helpful to you?“). Data collection is scheduled for summer 2025. Interviews will last approximately 45 to 60 minutes and will be conducted according to participants’ preferences – either in person, via Zoom, or by telephone. Remote sessions will be conducted using end-to-end encrypted Zoom connections, without using the platform's built-in recording function. All interviews will be audio-recorded using an external recording device. Transcription will be carried out by a transcription agency.
- *Focus group discussions*: The authors will develop the discussion guide for the evaluation in focus group discussions based on the experiences in the cultural adaptation and translation process. In the focus groups, participants will discuss the linguistic design and comprehensibility of the information (e.g. “How would you assess the terms and phrases used in terms of dementia-sensitive language?”), as well as critical passages from the cultural adaptation (e.g. “How would you assess the description of the types of dementia in terms of their precision and current status?”), the first draft of the DHL module (e.g. “What additional content would you like to see in this module?”), and possible designs for the interactive elements and implementations of previously text-based content in more appealing didactic formats (e.g. “How useful and relevant to everyday life did you find the examples and quiz questions?”). Data collection is scheduled for summer 2025. Each focus group will last approximately 60 to 90 minutes. The focus groups will be conducted at Ruhr University Bochum and will be audio-recorded using an external recording device.
- *Think-aloud sessions*: The think-aloud sessions will be conducted to assess the usability (including accessibility) of the *iSupport Deutschland* platform by capturing participants’ cognitive and behavioral responses during task-based interactions. In advance, sociodemographic data (e.g., year of birth, gender, occupation) will be collected using a standardized paper-based questionnaire. Informal caregivers will also complete the German version of GR-eHEALS [37] in this context. The sociodemographic questionnaire and GR-eHEALS will be administered in paper format. Participants will then be asked to verbalize their thoughts in a cognitive walkthrough while completing structured tasks designed to reflect typical usage scenarios and to uncover potential usability barriers. Verbalizations, gestures, facial expressions, and system interactions will be documented. Immediately following each cognitive walkthrough, a post-session interview will be conducted using a semi-structured interview guide. Interviews will be audio-recorded using an external recording device. After the post-session interviews, participants will be asked to complete the SUS [39] and the short version of the user experience questionnaire (UEQ-S) [40]. Participants may complete the questionnaires either independently or together with the researcher, depending on their preference. All completed questionnaires will be collected immediately following the post-session interview. For remote interviews, the questionnaires will be sent and returned by post. Data collection is scheduled for spring 2026. Each session will last approximately 60 min and will be conducted either in person or remotely via Zoom, depending on participant preference. Remote sessions will use end-to-end encrypted Zoom connections (without using the platform’s built-in recording function), with screen recordings captured locally on the researcher’s computer using the open-source software *OBS Studio* [41]. In all sessions, verbalizations and user interactions will be audio-recorded using an external recording device.

### Data handling

The contact details are only collected and stored for the purpose of contacting the study participants and for the payment of the expense allowance within the described project and within the described project duration.

Video and audio files as well as the data sets of the socio-demographic data and questionnaires of the informal caregivers and experts are pseudonymized so that an assignment to the contact data is no longer possible without the use of a coding list. The original documents of the questionnaires will be stored in a locked cabinet after digitization (scans) and pseudonymization until the end of the project. An external provider will transcribe the audio data verbatim. The transcribed and pseudonymized data will be organized using *MAXQDA* software [42] for qualitative analysis. The software *IBM SPSS Statistics* [36] will be used to analyze the quantitative data.

### Data analysis

The pseudonymized transcripts and screen recordings from the interviews, focus group discussions, and think-aloud sessions will be analyzed using a structuring content analysis [43]. Qualitative content analysis is the appropriate approach for the analysis of communicative content in a rule-based manner. An inductive-deductive approach is employed to explore the unknown field of research, while existing assumptions are examined on a case-by-case basis. The coding of the respective data will be carried out by two coders to ensure reliability, collegial validation, and intersubjective traceability.

The usability tests will be analyzed with the aim of capturing participants’ cognitive and emotional responses to the interaction design, content, and visual-aesthetic layout of the *iDEM-Support* platform. Quantitative data from the SUS, UEQ-S, and GR-eHEALS will be analyzed using descriptive statistics to characterize usability, user experience, and digital health literacy within the study sample. No inferential or subgroup analyses are planned.

### Ethics and consent to participate

Ethical approval for the study was obtained from the Ethics Committee of the Ruhr University Bochum, based in Bad Oeynhausen, Germany, in 2025 (Nr. 2025 − 1343).

Participation in the study requires written informed consent. In accordance with the principle of ongoing consent, participants are reminded during the study that their participation is voluntary. They can withdraw their consent at any time without giving reasons and without any disadvantages. While the study topics are not expected to pose inherent risks, participants may disclose sensitive information, potentially causing temporary emotional strain. Safeguarding measures will be implemented, and contact information for follow-up support will be provided. The study team will monitor adverse events continuously, with early termination considered if risks are higher than anticipated. Overall, anticipated risks are minimal and limited to short-term emotional reactions during the data collection, with no expected medium- or long-term burdens.

## Discussion

*iDEM-Support* will represent the first implementation of the WHO *iSupport* program in a German-language version tailored to the specific needs of informal caregivers of PlwD in Germany. The *iSupport* *Deutschland* platform will be a further refinement of *iSupport* by incorporating interactive features and a module on DHL, thereby enhancing its potential to empower informal caregivers. The study design reflects lessons learned from prior implementations in other countries [28–30, 33] and is shaped by a participatory development approach to ensure alignment with the target group’s needs and preferences.

The *iSupport* platform has demonstrated positive effects in previous studies, including improvements in emotional well-being, reductions in stress and burden, and a decreased risk of mental health problems among informal caregivers of PlwD [19, 20]. *iDEM-Support* aims to expand these outcomes through additional interactive and peer-exchange components. The platform’s digital nature ensures location-independent access, which is an important advantage for this target group. This aligns with the result of Etxeberria’s et al. (2021) study, which showed a high acceptance of digital interventions among informal caregivers [27]. A particular innovation of *iDEM-Support* is the development of a DHL module, enabling informal caregivers to find, understand, evaluate, and apply digital health information. Informed by established instructional design principles and evidence from the fields of health literacy and digital education, the module supports both knowledge acquisition and self-efficacy. Importantly, the module’s scope extends beyond dementia-specific content and caregiving roles, offering broader utility for patients in earlier stages of dementia and informal caregivers as well as relatives without caregiver role or professional caregivers. This approach ensures a high degree of user-centeredness, aiming for strong usability and a positive user experience [44], which should also be enabled by accessibility. Thus, the expected improvements in DHL can be a valuable contribution to public health by fostering the competent use of digital health information – in particular by maintaining the digital infrastructure of the *iSupport Deutschland* platform beyond the study period and thus ensuring long-term availability. The insights gained may inform similar adaptations in other national contexts. To ensure visibility and uptake within the scientific community, scientific results will be disseminated through presentations at academic conferences and publications in national and international journals.

Despite its expected strengths, the project has certain limitations. The recruitment strategy may favor informal caregivers who are more open to digital formats, potentially underrepresenting those with lower DHL or a fundamental lack of digital access. Nonetheless, the project contributes essential insights into user-centered design and the development of scalable, accessible digital interventions for informal caregivers – especially within a healthcare landscape increasingly shaped by digital transformation. Valuable findings are also likely to emerge from the subsequent proof of concept in the RCT.

*iDEM-Support* aims to address the critical gap in (self-)support for informal caregivers of PlwD. Using digital technologies, the study will provide valuable insights into the effects of internet-based information and communication technologies for informal caregivers of PlwD, including online training and support programs. Thus, the platform offers a promising way to reduce the burden on informal caregivers, thereby delaying the institutionalization of PlwD and strengthening informal care structures in an evolving digital healthcare landscape.

## Data Availability

Not applicable.

## References

[1] Federal Statistical Office of Germany. Ergebnisse der 14. koordinierten Bevölkerungsvorausberechnung. 2019. www.destatis.de/DE/Themen/Gesellschaft-Umwelt/Bevoelkerung/Bevoelkerungsvorausberechnung/Tabellen/variante-1-2-3-altersgruppen.html. Accessed 16 Jun 2025.

[2] World Health Organization. iSupport version 1.0. Adaptation and Implementation Guide. 2019. https://www.who.int/publications/i/item/9789241515863.

[3] Alzheimer’s Disease International. Dementia statistics. 2025. https://www.alzint.org/about/dementia-facts-figures/dementia-statistics/. Accessed 16 Jun 2025.

[4] Radtke R. Anzahl der Demenzkranken in Deutschland nach Alter und Geschlecht 2023. 2024. https://de.statista.com/statistik/daten/studie/246028/umfrage/anzahl-der-demenzkranken-in-deutschland-nach-alter-und-geschlecht/#statisticContainer. Accessed 16 Jun 2025.

[5] Blotenberg I, Thyrian JR. Die Häufigkeit von Demenzerkrankungen: Deutsche Alzheimer Gesellschaft e.V., Infoblatt 1. 2024.

[6] Yu Y, Xiao L, Ullah S, Meyer C, Wang J, Pot AM, Shifaza F. The experiences of informal caregivers of people with dementia in Web-Based psychoeducation programs: systematic review and metasynthesis. JMIR Aging. 2023;6:e47152. 10.2196/47152. 37247218 10.2196/47152 PMC10262022

[7] Wimo A, Gauthier S, Prince M. Global estimates of informal care. Alzheimer’s Disease International; 2018.

[8] Alzheimer’s Disease International. World Alzheimer Report 2009. 2009.

[9] Hellis E, Mukaetova-Ladinska EB. Informal caregiving and alzheimer’s disease: the psychological effect. Med (Kaunas). 2022. 10.3390/medicina59010048. 10.3390/medicina59010048 PMC9863258 36676672

[10] Riffin C, van Ness PH, Wolff JL, Fried T. Family and other unpaid caregivers and older adults with and without dementia and disability. J Am Geriatr Soc. 2017;65:1821–8. 10.1111/jgs.14910. 28426910 10.1111/jgs.14910 PMC5555780

[11] Gilhooly KJ, Gilhooly MLM, Sullivan MP, McIntyre A, Wilson L, Harding E, et al. A meta-review of stress, coping and interventions in dementia and dementia caregiving. BMC Geriatr. 2016;16:106. 10.1186/s12877-016-0280-8. 27193287 10.1186/s12877-016-0280-8 PMC4872341

[12] Sabatini S, Martyr A, Hunt A, Gamble LD, Matthews FE, Thom JM, et al. Health conditions in spousal caregivers of people with dementia and their relationships with stress, caregiving experiences, and social networks: longitudinal findings from the IDEAL programme. BMC Geriatr. 2024;24:171. 10.1186/s12877-024-04707-w. 38373905 10.1186/s12877-024-04707-w PMC10875834

[13] Afram B, Stephan A, Verbeek H, Bleijlevens MHC, Suhonen R, Sutcliffe C, et al. Reasons for institutionalization of people with dementia: informal caregiver reports from 8 European countries. J Am Med Dir Assoc. 2014;15:108–16. 10.1016/j.jamda.2013.09.012. 24238605 10.1016/j.jamda.2013.09.012

[14] German Center for Neurodegenerative Diseases e.V., German Alzheimer Society e.V., editors. Angehörige von Menschen mit Demenz: Forschungsergebnisse und Perspektiven. 1st ed. Weinheim, Basel: Beltz Juventa; 2025.

[15] Kuzuya M, Enoki H, Hasegawa J, Izawa S, Hirakawa Y, Shimokata H, Akihisa I. Impact of caregiver burden on adverse health outcomes in community-dwelling dependent older care recipients. Am J Geriatr Psychiatry. 2011;19:382–91. 10.1097/JGP.0b013e3181e9b98d. 20808120 10.1097/JGP.0b013e3181e9b98d

[16] Chang SJ, Yang E, Lee K-E, Ryu H. Internet health information education for older adults: A pilot study. Geriatr Nurs. 2021;42:533–9. 10.1016/j.gerinurse.2020.10.002. 33092906 10.1016/j.gerinurse.2020.10.002

[17] Pot AM, Blom MM, Willemse BM. Acceptability of a guided self-help internet intervention for family caregivers: mastery over dementia. Int Psychogeriatr. 2015;27:1343–54. 10.1017/S1041610215000034. 25648589 10.1017/S1041610215000034

[18] Schaeffer D, Gille S, Berens E-M, Griese L, Klinger J, Vogt D, Hurrelmann K. Digitale gesundheitskompetenz der Bevölkerung in deutschland: ergebnisse des HLS-GER 2. [Digital health literacy of the population in germany: results of the HLS-GER 2]. Gesundheitswesen. 2023;85:323–31. 10.1055/a-1670-7636. 34905785 10.1055/a-1670-7636 PMC11248737

[19] Hopwood J, Walker N, McDonagh L, Rait G, Walters K, Iliffe S, et al. Internet-Based interventions aimed at supporting family caregivers of people with dementia: systematic review. J Med Internet Res. 2018;20:e216. 10.2196/jmir.9548. 29895512 10.2196/jmir.9548 PMC6019848

[20] Teles S, Ferreira A, Paúl C. Assessing attitudes towards online psychoeducational interventions: psychometric properties of a brief attitudes scale. Health Soc Care Community. 2021;29:e1–10. 10.1111/hsc.13227. 33170537 10.1111/hsc.13227

[21] Link E, Baumann E. Nutzung von gesundheitsinformationen Im internet: personenbezogene und motivationale Einflussfaktoren. [Use of health information on the internet: personal and motivational influencing factors]. Bundesgesundheitsblatt Gesundheitsforschung Gesundheitsschutz. 2020;63:681–9. 10.1007/s00103-020-03144-5. 32367207 10.1007/s00103-020-03144-5 PMC8516774

[22] Sørensen K, van den Broucke S, Fullam J, Doyle G, Pelikan J, Slonska Z, Brand H. Health literacy and public health: a systematic review and integration of definitions and models. BMC Public Health. 2012;12:80. 10.1186/1471-2458-12-80. 22276600 10.1186/1471-2458-12-80 PMC3292515

[23] Norman CD, Skinner HA. eHealth literacy: essential skills for consumer health in a networked world. J Med Internet Res. 2006;8:e9. 10.2196/jmir.8.2.e9. 16867972 10.2196/jmir.8.2.e9 PMC1550701

[24] de Main AS, Xie B, Shiroma K, Yeh T, Davis N, Han X. Assessing the effects of eHealth tutorials on older adults’ eHealth literacy. J Appl Gerontology: Official J South Gerontological Soc. 2022;41:1675–85. 10.1177/07334648221088281. 10.1177/07334648221088281 PMC9232984 35466732

[25] Cramer A, Keinki C, Saur F, Walter S, Hübner J. eHealth literacy, internet and eHealth service usage: a survey among a German municipality. J Public Health (Berl). 2025;33:237–48. 10.1007/s10389-023-01997-z.

[26] Soares S, Hoffmeister LV, Fernandes MF, Henriques A, Costa A. The use of digital technologies in the promotion of health literacy and empowerment of informal caregivers: scoping review. JMIR Aging. 2024;7:e54913. 10.2196/54913. 38683655 10.2196/54913 PMC11091806

[27] Etxeberria I, Salaberria K, Gorostiaga A. Online support for family caregivers of people with dementia: a systematic review and meta-analysis of RCTs and quasi-experimental studies. Aging Ment Health. 2021;25:1165–80. 10.1080/13607863.2020.1758900. 32363901 10.1080/13607863.2020.1758900

[28] Teles S, Ferreira A, Paúl C. Feasibility of an online training and support program for dementia carers: results from a mixed-methods pilot randomized controlled trial. BMC Geriatr. 2022;22:173. 10.1186/s12877-022-02831-z. 35232389 10.1186/s12877-022-02831-z PMC8887647

[29] Baruah U, Varghese M, Loganathan S, Mehta KM, Gallagher-Thompson D, Zandi D, et al. Feasibility and preliminary effectiveness of an online training and support program for caregivers of people with dementia in india: a randomized controlled trial. Int J Geriatr Psychiatry. 2021;36:606–17. 10.1002/gps.5502. 33491811 10.1002/gps.5502

[30] Pinto-Bruno ÁC, Pot AM, Kleiboer A, Droes R-M, van Straten A. An online minimally guided intervention to support family and other unpaid carers of people with dementia: protocol for a randomized controlled trial. JMIR Res Protoc. 2019;8:e14106. 10.2196/14106. 31603433 10.2196/14106 PMC6819009

[31] Efthymiou A, Karpathiou N, Dimakopoulou E, Zoi P, Karagianni C, Lavdas M, et al. Cultural adaptation and piloting of iSupport dementia in Greece. Stud Health Technol Inf. 2022;289:184–7. 10.3233/SHTI210890. 10.3233/SHTI210890 35062123

[32] Molinari-Ulate M, Guirado-Sánchez Y, Platón L, van der Roest HG, Bahillo A, Franco-Martín M. Cultural adaptation of the iSupport online training and support programme for caregivers of people with dementia in Castilla y León. Spain Dement (London). 2023;22:1010–26. 10.1177/14713012231165578. 10.1177/14713012231165578 PMC10262323 36942726

[33] Xiao LD, Ye M, Zhou Y, Rita Chang H-C, Brodaty H, Ratcliffe J, et al. Cultural adaptation of world health organization iSupport for dementia program for Chinese-Australian caregivers. Dement (London). 2022;21:2035–52. 10.1177/14713012221110003. 10.1177/14713012221110003 35724375

[34] Messina A, Amati R, Annoni AM, Albanese E, Fiordelli M. iSupport swiss: a community based participatory approach to culturally adapt the WHO online intervention for family caregivers of people with dementia. Int Psychogeriatr. 2023;35:59. 10.1017/S1041610224000152.36541077

[35] Milanti A, Norman C, Chan DNS, So WKW, Skinner H. eHealth literacy 3.0: updating the Norman and Skinner 2006 model. J Med Internet Res. 2025;27:e70112. 10.2196/70112. 40068166 10.2196/70112 PMC11937704

[36] IBM Corp. IBM SPSS Statistics for Windows; 2024.

[37] Marsall M, Engelmann G, Skoda E-M, Teufel M, Bäuerle A. Measuring electronic health literacy: Development, Validation, and test of measurement invariance of a revised German version of the eHealth literacy scale. J Med Internet Res. 2022;24:e28252. 10.2196/28252. 35107437 10.2196/28252 PMC8851340

[38] Norman CD, Skinner HA. eHEALS: the eHealth literacy scale. J Med Internet Res. 2006;8:e27. 10.2196/jmir.8.4.e27. 17213046 10.2196/jmir.8.4.e27 PMC1794004

[39] Rummel B. System Usability Scale – jetzt auch auf Deutsch. 2016. https://blogs.sap.com/2016/02/01/system-usability-scale-jetzt-auch-auf-deutsch/.

[40] Schrepp M, Thomaschewski J, Hinderks A. Design and evaluation of a short version of the user experience questionnaire (UEQ-S). Int J Interact Multimedia Artif Intell. 2017;4:103–8. 10.9781/ijimai.2017.09.001.

[41] Open Broadcaster Studio. OBS Studio; 2025.

[42] VERBI GmbH. MAXQDA; 2025.

[43] Kuckartz U, Rädiker S. Qualitative Inhaltsanalyse - Methoden, Praxis, umsetzung Mit software und künstlicher intelligenz. 6th ed. Weinheim, Basel: Beltz Juventa; 2024.

[44] Karpathakis K, Libow G, Potts HWW, Dixon S, Greaves F, Murray E. An evaluation service for digital public health interventions: User-Centered design approach. J Med Internet Res. 2021;23:e28356. 10.2196/28356. 34494965 10.2196/28356 PMC8459216

